# Patient experience with hereditary and senile systemic amyloidoses: a survey from the Amyloidosis Research Consortium

**DOI:** 10.1186/1750-1172-10-S1-P22

**Published:** 2015-11-02

**Authors:** Isabelle Lousada, Raymond L Comenzo, Heather Landau, Spencer Guthrie, Giampaolo Merlini

**Affiliations:** 1 Amyloidosis Research Consortium, Inc., N/A, 01773, Lincoln, USA; 2 Tufts Medical Center, N/A, 02111, Boston, USA; 3 Memorial Sloan-Kettering Cancer Center, N/A, 10065, New York, USA; 4 Prothena Biosciences Inc, N/A, 94080, South San Francisco, USA; 5 Fondazione IRCCS Policlinico San Matteo, and University of Pavia, N/A, 27100, Pavia, Italy

## Background

Amyloidosis is caused by the accumulation of misfolded proteins, resulting in dysfunction of vital organs (eg, heart, kidneys, nervous system). Diagnosis and access to appropriate therapy pose significant challenges, and there is a paucity of literature depicting the patient (pt) experience. We conducted a survey to identify the challenges in establishing a diagnosis of amyloidosis and to gain insight into the pt experience.

## Methods

Pts with any type of systemic amyloidosis and their family members and caregivers were invited to participate in an anonymous online survey through email and social media channels of the Amyloidosis Foundation and an amyloidosis awareness group on Facebook. Caregivers and family members could answer on behalf of pts. The 16-question survey was developed by the authors and was available to participants online from January 29 to February 5, 2015. Here we present survey results focusing on pts with hereditary and senile systemic amyloidosis (SSA).

## Results

In total, 533 persons completed the survey (pts, 58%; family members, 34%; caregivers, 8%). Overall, most pts were female (62%) and between 50 and 69 years of age (62%) (average age at diagnosis, 57 years). Most pts (72%) had light chain (AL) amyloidosis. Hereditary forms of amyloidosis included transthyretin (TTR)-related amyloidosis (ATTR; n=37) and hereditary non-TTR amyloidosis (n=18); SSA/wild-type ATTR was reported by 13 patients. 57%, 33%, and 77% of pts with ATTR, non-TTR, and wild-type ATTR were male, and average age at diagnosis was 54, 55, and 71 years, respectively. The most frequently involved organs were heart and nervous system in pts with ATTR, kidney and nervous system in pts with non-TTR, and heart in pts with wild-type ATTR. In pts with ATTR, non-TTR, and wild-type ATTR, the correct diagnosis was made within 6 months in 35%, 22%, and 46% of pts, respectively. For pts with ATTR, the correct diagnosis was most commonly made by a cardiologist (24%), neurologist (22%), or primary care physician (11%). For those with non-TTR, the correct diagnosis was most commonly made by a hematologist (22%), nephrologist (17%), or specialist at an amyloidosis center (17%). Pts with wild-type ATTR most commonly received the diagnosis from cardiologists (62%). Across groups, less than one-third of pts knew how to enroll in a clinical trial, but half would consider participating if more informed.

## Conclusions

A correct diagnosis of hereditary or wild-type ATTR amyloidosis often requires numerous physician visits to different medical specialists and often occurs when disease is advanced. Responses obtained in this survey highlight the challenges experienced by pts with these rare diseases. These data may identify opportunities to educate pts and physicians in order to expedite diagnosis, facilitate appropriate disease management and access to clinical trials, and ultimately improve pt survival.

